# Rotundic Acid Protects against Metabolic Disturbance and Improves Gut Microbiota in Type 2 Diabetes Rats

**DOI:** 10.3390/nu12010067

**Published:** 2019-12-26

**Authors:** Zenghao Yan, Hao Wu, Hongliang Yao, Wenjun Pan, Minmin Su, Taobin Chen, Weiwei Su, Yonggang Wang

**Affiliations:** 1 State Key Laboratory of Biocontrol and Guangdong Provincial Key Laboratory of Plant Resources, School of Life Sciences, Sun Yat-Sen University, Guangzhou 510275, China; yanzengh@mail2.sysu.edu.cn (Z.Y.); wuhao8@mail.sysu.edu.cn (H.W.); yaohl@giabr.gd.cn (H.Y.); panwj23@mail2.sysu.edu.cn (W.P.); tagorem@163.com (M.S.); sysulssctb@126.com (T.C.); lsssww@126.com (W.S.); 2 Shenzhen Research Institute of Sun Yat-Sen University, Shenzhen 518057, China; 3 Guangdong Key Laboratory of Animal Conservation and Resource Utilization, Guangdong Public Laboratory of Wild Animal Conservation and Utilization, Drug Synthesis and Evaluation Center, Guangdong Institute of Applied Biological Resources, Guangzhou 510260, China

**Keywords:** rotundic acid, type 2 diabetes, gut microbiota, metabolic disturbance, diabetic complications

## Abstract

Rotundic acid (RA) is a major triterpene constituent in the barks of *Ilex rotunda* Thunb, which have been widely used to make herbal tea for health care in southern China. RA has a variety of bioactivities such as anti-inflammation and lipid-lowering effect. However, little is known about the effects and mechanisms of RA on metabolic disturbance in type 2 diabetes (T2D) and its effect on gut microbiota. A T2D rat model induced by high fat diet (HFD) feeding and low-dose streptozotocin (STZ) injection was employed and RA showed multipronged effects on T2D and its complications, including improving glucolipid metabolism, lowering blood pressure, protecting against cardiovascular and hepatorenal injuries, and alleviating oxidative stress and inflammation. Furthermore, 16s rRNA gene sequencing was carried out on an Illumina HiSeq 2500 platform and RA treatment could restore the gut microbial dysbiosis in T2D rats to a certain extent. RA treatment significantly enhanced the richness and diversity of gut microbiota. At the genus level, beneficial or commensal bacteria *Prevotella*, *Ruminococcus*, *Leuconostoc* and *Streptococcus* were significantly increased by RA treatment, while RA-treated rats had a lower abundance of opportunistic pathogen *Klebsiella* and *Proteus*. Spearman’s correlation analysis showed that the abundances of these bacteria were strongly correlated with various biochemical parameters, suggesting that the improvement of gut microbiota might help to prevent or attenuate T2D and its complication. In conclusion, our findings support RA as a nutraceutical agent or plant foods rich in this compound might be helpful for the alleviation of T2D and its complications through improving gut microbiota.

## 1. Introduction

Type 2 diabetes (T2D), which is characterized by low-grade inflammation, insulin resistance and pancreatic β-cell dysfunction, has become a major public health issue worldwide [1]. The prevalence of diabetes among adults was estimated to be 8.4% in 2017 and predicted to rise to 9.9% in 2045 [2], and over 90% of diabetic patients suffer from T2D [3]. The development of T2D results mostly from obesity, which is associated with a high-calorie dietary style and other unhealthy living habits [4]. In particular, T2D induces severe metabolic disorders and vascular diseases, such as hyperlipemia, hypertension, and cardiovascular and cerebrovascular diseases, which together contribute to high rates of morbidity and mortality [5].

Emerging evidence suggests that gut microbiota plays a vital role in intestinal immunity, host metabolism and the development of various diseases, including T2D and diabetic complications. T2D patients have a moderate degree of gut microbial dysbiosis. A metagenome-wide association study of fecal samples of 171 T2D patients and 174 healthy controls showed that diseased samples had a decrease in the abundance of some universal butyrate-producing bacteria and an increase in various opportunistic pathogens [6]. The gut microbial compositions in T2D patients with coronary artery diseases were significantly associated with the increase in the serum level of trimethylamine oxide, which has been suggested to be a microbiota-dependent metabolite to mediate the development of T2D [7,8]. Furthermore, more and more optimizing strategies, especially nutraceutical agents and foods, have been taken to target gut microbiota to improve health and attenuate diseases. For instance, a high-fiber diet containing traditional Chinese medicinal foods such as tartary buckwheat and adlay induced changes in the entire gut microbial community and alleviated T2D via the production of short-chain fatty acids (SCFAs) [9]. For the development of nutraceutical food, these studies suggest that gut microbiota may be a door opened to the prevention and alleviation of T2D and diabetic complications.

Rotundic acid (RA) is a major triterpene constituent in *Ilex rotunda* Thunb., and the barks of *Ilex rotunda* Thunb. are widely used to make herbal tea for health care in southern China. RA has a variety of bioactivities including anti-inflammation and significant cytotoxicity activities in the Daoy, Hep-2 and MCF-7 cells (human tumor cell lines) in vitro [10]. A study also showed that RA decreased plasma triglyceride and cholesterol levels and declined cardiac and renal levels of tumor necrosis factor-α (TNF-α) and monocyte chemoattractant protein-1 in streptozotocin-induced type 1 diabetes mice [11]. However, little is known about the effects and mechanisms of RA on metabolic disturbance in T2D. On the other hand, microbial metabolism is capable of metabolizing RA into bioactive metabolites [12]. Due to the chemical complexity, RA may be hard to be absorbed in the small intestine and then pile up in the lumen of the large intestine in the intact form [13]. We hypothesized that RA could improve gut microbiota to protect against metabolic disturbance in T2D.

In this study, Sprague–Dawley rats were fed with high-fat diet (HFD) followed by a single low-dose streptozotocin (STZ) injection to induce T2D. The T2D rat model was employed to investigate the protective effects of RA on metabolic disturbance in T2D and 16s rRNA gene analysis of fecal samples was also carried out. The aims of this study were to evaluate the protective effects of RA against T2D and its complications and to provide new insight into the underlying mechanisms through improving gut microbiota.

## 2. Materials and Methods

The animal protocols in this study were supervised and approved by the Ethics Committee of School of Life Sciences, Sun Yat-sen University in June 2017 (0017113). All animal experiments were carried out in strict accordance with the Guide for the Care and Use of Laboratory Animals published by National Institutes of Health (NIH). Animals were housed in a standardized pathogen-free area with ambient temperature (23 ± 3 °C), humidity (55 ± 15%) control and a 12 h light/dark cycle (animal use certificate No. SYXK (yue) 2014-0020). Every effort was made to minimize both the employed animal numbers and the potential discomfort of the animals throughout the study.

### 2.1. Chemicals, Animals and Diets

The sodium salt of rotundic acid (RA) (98%, CAS Number: 20137-37-5) was prepared from the barks of *Ilex rotunda* Thunb. (produced in Guangxi, Kangsheng Medical Company, Guangzhou, China) and verified by RA standard (Shenzhen Phystandard Biological Technology Co., Ltd., Shenzhen, China). Some details and data about the verification of RA prepared in this study were provided in Supplementary Materials (Figure S1). The RA preparation steps included ethanol reflux extraction, activated carbon adsorption, sodium hydroxide hydrolysis and recrystallization. Streptozotocin (STZ) and d-(+)-glucose were purchased from Sigma-Aldrich Co. (St. Louis, MO, USA). Insulin was purchased from Novo (China) Pharmaceutical Co., Ltd. (Beijing, China). The 5-week-old male Sprague–Dawley rats were purchased from Guangdong Medical Laboratory Animal Center (Laboratory animal quality certificate No. 44007200040426, Foshan, China). The rodent standardized diet was purchased from Guangdong Medical Laboratory Animal Center (Foshan, China). The rodent high-fat diet (HFD) with 60 kcal% fat was purchased from Research Diets Inc. (D12492, New Burnswick, NJ, USA).

### 2.2. Experimental Design

After one week of adaptation with general meals and diet balance, 30 rats with body weights from 180 to 220 g were randomly divided into a control group (*n* = 10) and HFD group (*n* = 20) and fed with standardized diet and HFD for a continuous 10 weeks, respectively. In this 10 week HFD period, after 12 h fasting, HFD rats were intraperitoneally injected with low-dose STZ (dissolved in 30 mg/kg in ice cold citrate buffer, pH = 4.5) to induce the T2D model at the end of week 8 (day 54). The control rats were injected with an equal volume of citrate buffer (ice cold, pH = 4.5). Then, 72 h post injection, fast blood glucose levels were measured by OneTouch^®^ UltraVue glucometer (Johnson & Johnson Services, Inc., New Burnswick, NJ, USA), and HFD rats with fast blood glucose levels no less than 11.0 mM were considered to suffer from T2D and recruited for further experiments. To evaluate the effects of RA, we randomly divided the T2D rats into the model group and RA-treated group (*n* = 8 per group). The treatments were administrated daily for an additional 8 weeks as follows: (1) control group: standardized diet with saline; (2) model group: HFD with saline; (3) RA-treated group: HFD with RA (40 mg/kg/day, dissolved in saline). RA at a dose of 40 mg/kg/day was administrated by gavage daily and all the groups received an equal volume of saline. Throughout the study, rats were fed ad libitum and their body weights were monitored weekly. The amounts of remaining diet and water were weighted every week to determine the food and water intake of the rats.

### 2.3. The Oral Glucose Tolerance Test (OGTT) and the Insulin Tolerance Test (ITT)

Oral glucose tolerance and insulin tolerance were measured in week 18. For the OGTT, rats were challenged with 3.0 g/kg of glucose intragastrically after 12 h fasting. Then, blood samples were collected at 0, 15, 30, 45, 60, 90, 120 min post administration via tail vein. For the ITT, rats were injected with 0.75 IU/kg of insulin intraperitoneally after 4h fasting. Blood samples were collected at 0, 15, 30, 45, 60, 90, 120 min post injection via tail vein. The blood glucose levels were measured by OneTouch^®^ UltraVue glucometer (Johnson & Johnson Services, Inc., New Burnswick, NJ, USA).

### 2.4. Measurement of Blood Pressure

The blood pressures of rats were measured on day 126 by indirect tail-cuff method with BP-2010A system (Softron Inc., Tokyo, Japan). The systolic blood pressure (SYS), diastolic blood pressure (DIA) and mean arterial pressure (MAP) of each rat were measured three times.

### 2.5. Blood/Serum Biochemical Analysis

Rats were sacrificed after 12 h fasting on day 127 and blood/serum samples were collected and stored at −80 °C for biochemical analysis. The glycated hemoglobin A1c (HbA1c) levels were measured in whole blood samples using a HbA1c assay kit (Shanghai Kehua Bio-engineering Co., Ltd., Shanghai, China) and an automatic biochemical analyzer (7020, Hitachi, Ltd., Tokyo, Japan). The levels of glucose (GLU), total glyceride (TG), total cholesterol (TC), high-density lipoprotein cholesterol (HDL-C), low-density lipoprotein cholesterol (LDL-C), free fatty acids (FFAs), α-hydroxybutyrate dehydrogenase (α-HBDH), creatine kinase (CK), creatine kinase isoenzyme (CK-MB), lactate dehydrogenase (LDH), superoxide dismutase (SOD), malondialdehyde (MDA), alanine aminotransferase (ALT), aspartate aminotransferase (AST), alkaline phosphatase (ALP), uric acid (UA), creatinine (CRE), and urea nitrogen (BUN) in serum samples were measured and quantified by colorimetric kits (Nanjing Jiancheng Bio-engineering Institute, Nanjing, China) according to the manufacturer’s instructions. The levels of interleukin-1β (IL-1β), interleukin-4 (IL-4), interleukin-6 (IL-6), TNF-α, interferon-γ (INF-γ), insulin (INS), angiotensin-2 (ANG-2), C reactive protein (CRP), and endothelin-1 (ET-1) in serum samples were measured and quantified by ELISA kits (Cloud-Clone Corp., Katy, TX, USA) according to the manufacturer’s instructions. The HOMA-IR index was calculated by the following equation: [glucose (mmol/L) × insulin (mIU/L)]/22.5.

### 2.6. Fecal DNA Extraction

On day 126, fecal samples were collected from each rat and frozen at −80 °C until further use. The PowerSoil^®^ DNA isolation kit (MO BIO Laboratories, Inc., Carlsbad, CA, USA) was used for fecal DNA extraction according to the manufacturer’s instructions. The integrity of extracted DNA was verified by 1% agarose gel electrophoresis.

### 2.7. V4–V5 16s rRNA Gene Region Amplification and Sequencing

The hypervariable V4–V5 regions of the 16S rRNA genes from bacteria and archaea were PCR amplified in triplicate using the barcoded primers 515-F (5′-GTGCCAGCMGCCGCGGTAA-3′) and 907-R (5′-CCGTCAATTCMTTTRAGTTT-3′). PCR amplifications were performed with the following thermocycling conditions: 94 °C for 5 min followed by 30 cycles of 94 °C for 30 s, 52 °C for 30 s, and 72 °C for 30 s and a final extension at 72 °C for 10 min. The PCR products were verified by 1% agarose gel electrophoresis and quantified using GeneTools Analysis Software (Version 4.03.05.0, SynGene, Frederick, MD, USA). Technical triplicate amplicons were pooled and gel purified using the EZNA Gel Extraction Kit (Omega Bio-tek, Inc., Norcoross, GA, USA). A gene library was constructed using the NEBNext^®^ Ultra™ DNA Library Prep Kit for Illumina^®^ (New England Biolabs, Ipswich, MA, USA). Sequencing was performed on an Illumina HiSeq 2500 platform (Illumina, Inc., San Diego, CA, USA) according to the manufacturer’s instructions and 250 bp paired-end reads were generated.

### 2.8. Sequencing Data Analysis

The paired-end raw reads were de-multiplexed and filtered according to the barcoded primers using Trimmomatic software (V 0.33, http://www.usadellab.org/cms/?page=trimmomatic) and Mothur software (V 1.35.1, http://www.mothur.org). Reads with an average quality score lower than 20, ambiguous base calls, primer mismatches or shorter than 100 bp were excluded from the analysis in order to increase the level of accuracy. After the quality filter, sequence assembly was performed using FLASH software (V 1.2.11, https://ccb.jhu.edu/software/FLASH/) and quality control was carried out using Mothur software. The operational taxonomic units (OTUs) were selected by clustering sequences with a similarity of >97% using Usearch algorithm (http://www.drive5.com/usearch/) [14] and the representative sequences were screened as the most abundant in each cluster using the Quantitative Insights into Microbial Ecology (QIIME) 1.8.0 software (http://qiime.org) [15]. OTUs were annotated with taxonomic information based on the RDP classifier version 2.2 algorithm using the Greengenes 16S rRNA gene database [16]. QIIME 1.8.0 software was also used to evaluate alpha and beta diversity, as described.

### 2.9. Statistical Analysis

Statistical analysis was performed using SPSS statistical software (Version 22.0, SPSS Inc., Chicago, IL, USA), GraphPad Prism 7 (San Diego, CA, USA) and R (version 3.5.2). The data were presented as the mean ± SD. Differences among groups were analyzed using one-way ANOVA followed by Tukey’s multiple comparison test only when the data was normal and the variances were equal. Otherwise, the Kruskal–Wallis test and the Mann–Whitney test were applied. A *p* value of < 0.05 was considered statistically significant. Spearman’s correlation analysis was performed to identify the correlations.

## 3. Results

### 3.1. Effect of RA on Body Weight, Water Intake and Food Intake in the Rats with T2D

**Figure 1:**
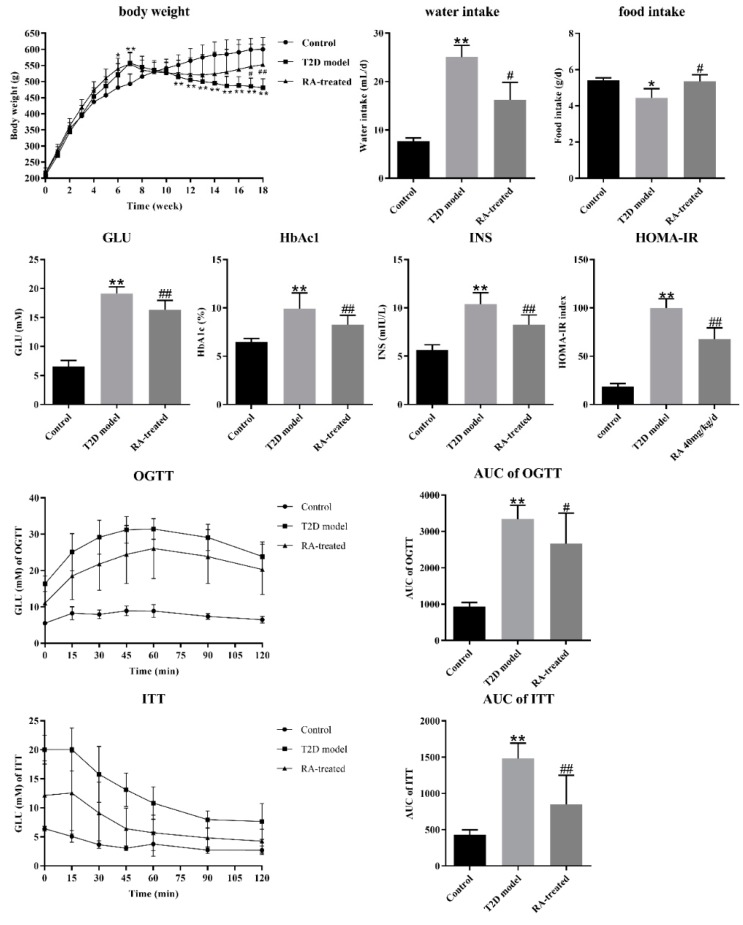
Effects of rotundic acid (RA) on body weight, water intake, food intake and glucose metabolism (glucose (GLU), HbAc1, insulin (INS), the HOMA-IR index, the oral glucose tolerance test (OGTT), the area under the curve (AUC) of the OGTT, the insulin tolerance test (ITT) and the AUC of the ITT). Data were presented as the mean ± SD (*n* = 8 per group). * *p* < 0.05 vs. control group; ** *p* < 0.01 vs. control group; # *p* < 0.05 vs. T2D model group; ## *p* < 0.01 vs. T2D model group by Tukey’s multiple comparison test.

As shown in Figure 1, the T2D model group dramatically increased in body weight compared with the control group after 6 weeks of HFD feeding (*p* < 0.05) and the STZ injection caused an expected decrease in body weight (*p* < 0.01), while at the end of this experiment, the RA-treated group had an increased body weight compared with the T2D model group (*p* < 0.01). In addition, RA treatment decreased water intake and increased food intake in the T2D rats when compared with the T2D model group (*p* < 0.05) in the last week of the whole experimental period (week 18).

### 3.2. RA Improved Glycemic Control and Insulin Resistance in the Rats with T2D

As shown in Figure 1, the T2D model group exhibited significantly elevated GLU, HbA1c and INS levels compared with the control group (*p* < 0.01), whereas RA treatment reduced GLU, HbA1c and INS levels in T2D rats (*p* < 0.01). The results of the OGTT, ITT and HOMA-IR index are important indices in the assessment of insulin resistance. According to the OGTT and ITT, blood glucose levels significantly increased before and after glucose and insulin load in the T2D model group compared with the control group (*p* < 0.01). The elevated glucose areas under the curve (AUCs) of the OGTT and ITT and HOMA-IR index in the T2D model group indicated severe glucose intolerance and insulin resistance in the rats with T2D (*p* < 0.01, Figure 1). When compared with the T2D model group, RA treatment significantly reduced blood glucose levels before and after glucose and insulin load in the rats with T2D (*p* < 0.01). Similarly, the rats with T2D treated with RA exhibited a reduced glucose AUC of the OGTT and ITT and HOMA-IR index (*p* < 0.01).

### 3.3. RA Improved Serum Lipid Profiles in the Rats with T2D

**Figure 2:**
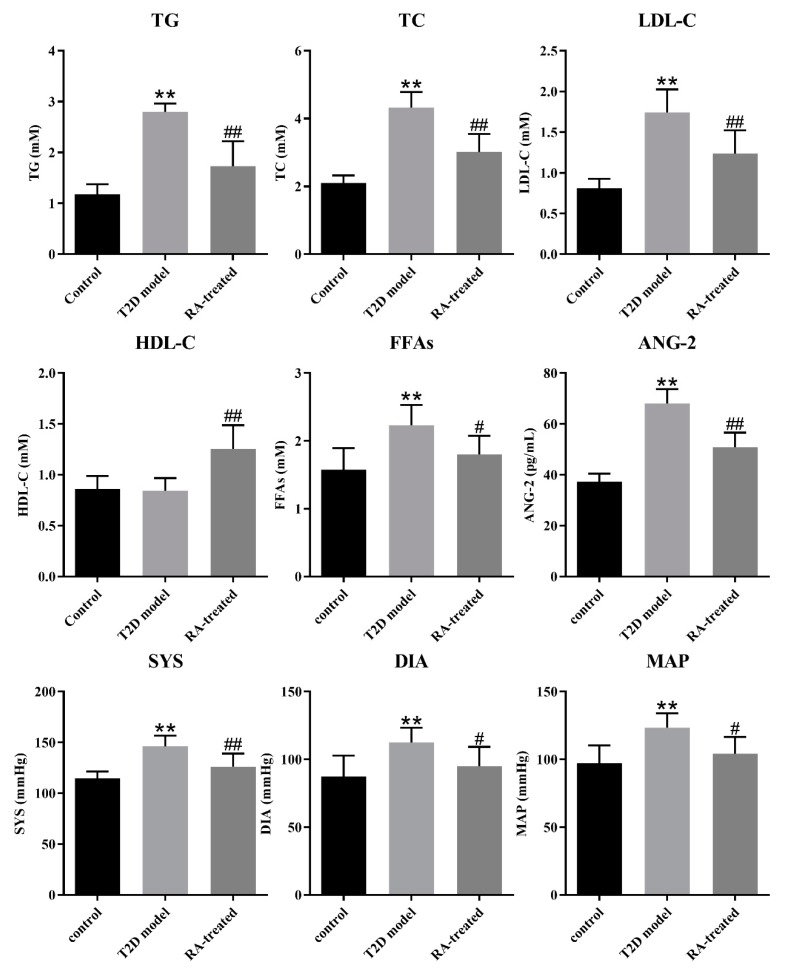
Effects of RA on lipid metabolism (total glyceride (TG), total cholesterol (TC), low-density lipoprotein cholesterol (LDL-C), high-density lipoprotein cholesterol (HDL-C) and free fatty acids (FFAs)) and blood pressure (angiotensin-2 (ANG-2), systolic blood pressure (SYS), diastolic blood pressure (DIA) and mean arterial pressure (MAP)). Data were presented as the mean ± SD (*n* = 8 per group). ** *p* < 0.01 vs. control group; # *p* < 0.05 vs. T2D model group; ## *p* < 0.01 vs. T2D model group by Tukey’s multiple comparison test.

As shown in Figure 2, serum TG, TC, LDL-C and FFA levels markedly increased in the T2D model group compared with the control group (*p* < 0.01). In contrast, increases in the serum TG, TC, LDL-C and FFA levels of the rats with T2D were significantly inhibited by RA treatment (*p* < 0.05). In addition, RA treatment significantly increased the HDL-C levels compared with the T2D model group (*p* < 0.05).

### 3.4. RA Lowered Blood Pressure in the Rats with T2D

Figure 2 indicates that there were statistical increases in blood pressure levels (SYS, DIA and MAP) in the T2D model group compared with the control group (*p* < 0.01) and the elevated blood pressure levels could be controlled by RA treatment (*p* < 0.05). ANG-2 is a key protein in the renin–angiotensin system to regulate blood pressure. As shown in Figure 2, RA treatment significantly decreased the serum level of ANG-2 in the rats with T2D compared with the T2D model group.

### 3.5. Protective Effects of RA on Cardiovascular, Hepatic and Renal Injuries in the Rats with T2D

Serum myocardial enzymes (α-HBDH, CK, CK-MB and LDH), CRP and ET-1 levels were always evaluated as key indicators of cardiovascular injury. As shown in Table 1, serum myocardial enzymes, CRP and ET-1 levels of the T2D model group were significantly higher than those of the control group (*p* < 0.01). In addition, the serum levels of ALT, AST, ALP, UA, BUN and CRE were also significantly elevated in the T2D model group compared with the control group (*p* < 0.01), which suggested hepatic and renal functions of the rats with T2D were impaired with the development of T2D. Changes in these indicators were significantly inhibited by RA treatment (*p* < 0.05), indicating the protective effect of RA on cardiovascular, hepatic and renal injury.

### 3.6. RA Alleviated Oxidative and Inflammatory Stress in the Rats with T2D

To investigate the protective effect of RA on oxidative and inflammatory stress in the rats with T2D, we determined the serum levels of various oxidative stress factors (SOD, MDA) and cytokines (TNF-α, INF-γ, IL-1β, IL-4, and IL-6). As shown in Table 2, the serum levels of MDA, IL-1β, TNF-α, INF-γ and IL-6 significantly increased while the serum levels of SOD and IL-4 markedly decreased in the T2D model group compared with the control group (*p* < 0.01). RA treatment increased the serum levels of SOD and IL-4 and decreased the serum levels of MDA, IL-1β, TNF-α, INF-γ and IL-6 in the rats with T2D, showing RA treatment could alleviate oxidative and inflammatory stress with the development of T2D.

### 3.7. Overall Structural Modulation of Gut Microbiota after RA Treatment

**Figure 3:**
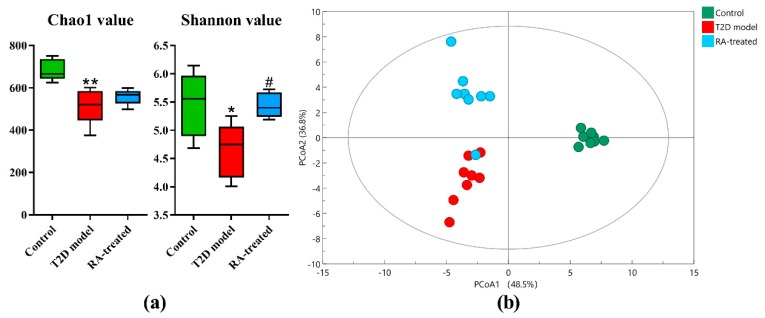
(**a**) The α-diversity of the Chao1 value and Shannon value for the control, T2D model and RA-treated groups. * *p* < 0.05 vs. control group; ** *p* < 0.01 vs. control group; # *p* < 0.05 vs. T2D model group by the Kruskal–Wallis test. (**b**) Principal coordinate analysis (PCoA) based on weighted unifrac metrics of the microbial community for the control, T2D model and RA-treated groups.

A total of 718,211 high-quality reads were acquired from the 24 fecal samples, with an average of approximate 29,925 sequences for each sample. After quality and chimera checking, 683 OTUs were determined (with 97% similarity). The Chao1 value and Shannon value were calculated to estimate the α-diversity. As shown in Figure 3a, the averages of the Chao1 value and Shannon value of each group suggested a significant decrease in microbial richness and diversity in fecal samples of the T2D model group in comparison with the control group (*p* < 0.05), and RA treatment had a tendency of increasing these parameters. Principal coordinate analysis (PCoA) plots based on weighted unifrac metrics were carried out to compare the β-diversity of gut microbiota among tested groups. Figure 3b shows a distinct separation among three groups and the PCoA accounted for 85.3% of gut microbiota profiles. These results indicated that the gut microbiota of T2D rats was different from the healthy rats, and the difference included both a significant decrease in microbial richness and diversity and a significant shift in the microbial community composition. According to α- and β-diversity analysis, RA treatment significantly enhanced the richness and diversity of gut microbiota and modulated the microbial community composition in T2D rats.

### 3.8. Relative Abundances of Different Bacteria in the Gut Microbiota after RA Treatment

**Figure 4:**
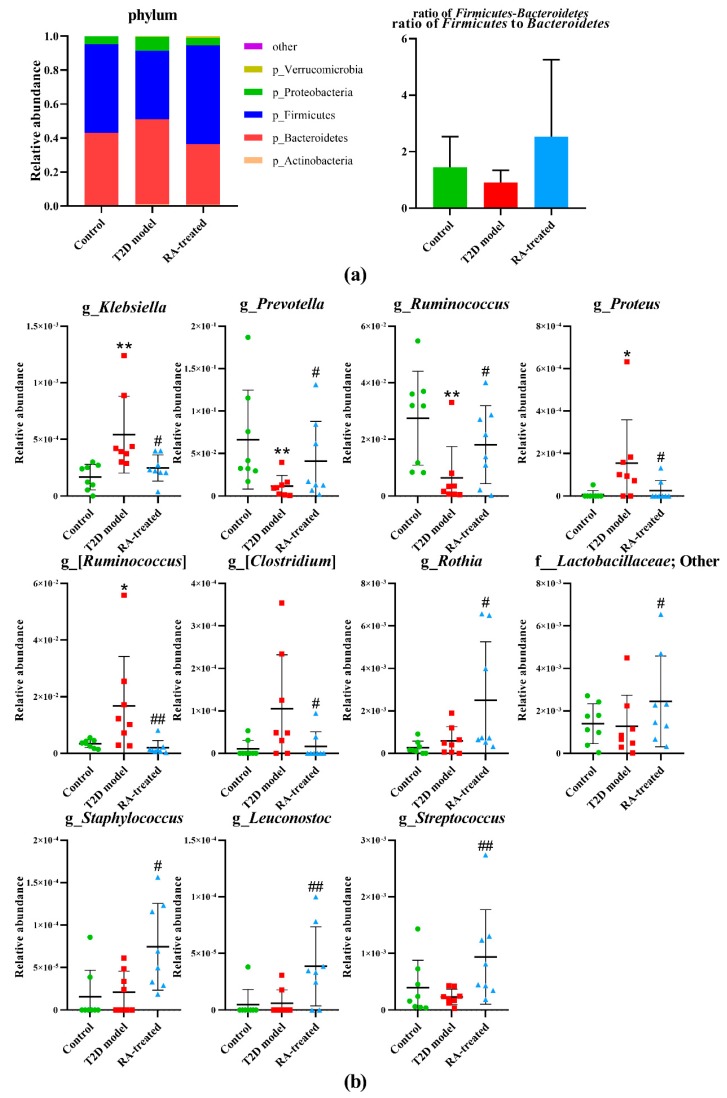
(**a**) Composition analysis of gut microbiota at the phylum level in the control, T2D model and RA-treated groups. (**b**) Relative abundances of gut microbiota at the genus level in the control, T2D model and RA-treated groups. * *p* < 0.05 vs. control group; ** *p* < 0.01 vs. control group; # *p* < 0.05 vs. T2D model group; ## *p* < 0.01 vs. T2D model group by Mann–Whitney U rank sum test.

**Figure 5:**
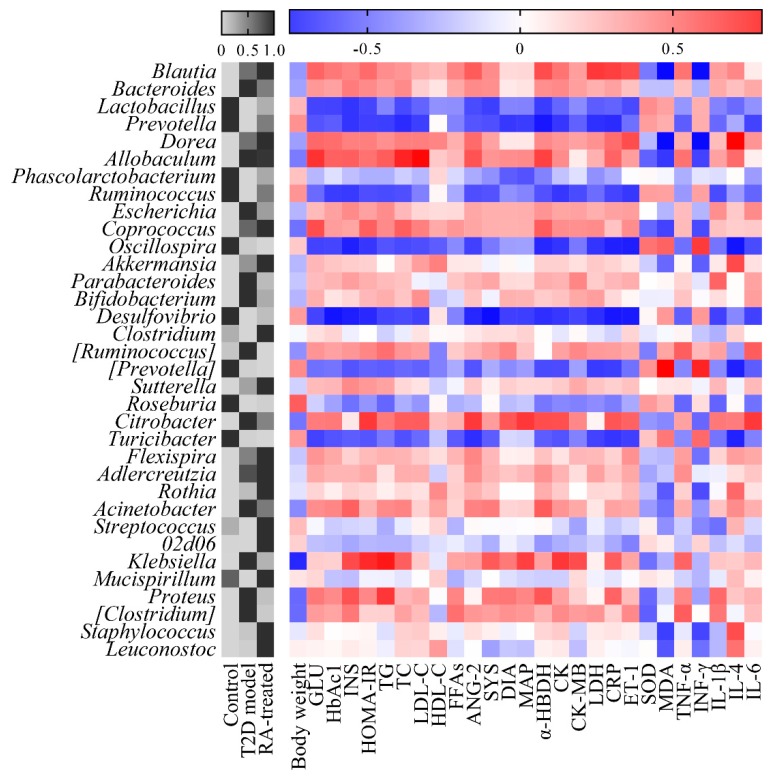
The heatmap of relative abundances of major genera in fecal samples of different groups are presented in gray levels after min–max normalization (**left**) and Spearman’s correlation value between relative abundances of major genera and biochemical parameters (**right**).

In this study, variations in the composition of gut microbiota among tested groups were observed at different bacterial levels. At the phylum level, the primary phylum were Firmicutes and Bacteroidetes in all samples (Figure 4a). Figure 4a also showed that the T2D model group had an elevated ratio of Firmicutes to Bacteroidetes and that this tendency was reversed by RA treatment in T2D rats. The relative abundances of major genera in fecal samples of different groups are presented as gray levels in Figure 5 after min–max normalization. Significant differences in microbial biomarkers among the tested groups were determined using the Mann–Whitney U rank sum test. At the genus level, the f_*Rikenellaceae*;g_, *Paraprevotella*, *Anaerofilum*, *Oscillospira*, [*Prevotella*], *Turicibacter*, *Dehalobacterium*, *Desulfovibrio*, *Prevotella*, *AF12*, f_*Peptococcaceae*;g_, *Ruminococcus*, o_*RF39*;f_;g_, and o_*YS2*;f_;g_ abundances were highly significantly reduced in the T2D model group compared to the control group, whereas the *Blautia*, *Allobaculum*, *Klebsiella*, *Christensenella*, f_*Lachnospiraceae*, other, *Dorea*, *Butyricicoccus*, *Citrobacter*, *Coprococcus*, and *Clostridium* abundances were highly significantly elevated (*p* < 0.01). As shown in Figure 4b, when compared with the T2D model group, the *Klebsiella*, *Prevotella*, *Ruminococcus*, *Proteus*, [*Ruminococcus*], [*Clostridium*], *Rothia*, f_*Lactobacillaceae*, other, *Staphylococcus*, *Leuconostoc*, and *Streptococcus* abundances were significantly regulated by RA treatment (*p* < 0.05).

### 3.9. Correlations between Microbial Abundances and Biochemical Biomarkers

Spearman’s correlation analysis was carried out including all tested samples to determine the relationships between microbial abundances at the genus level and biochemical parameters. As shown in Figure 5, the relative abundances of multiple genera were found to have significant correlations with various biochemical parameters. Among these, the Prevotella abundance was strongly negatively correlated with INS, TC, α-HBDH, CK, LDH and CRP, and strongly positively correlated with SOD (*p* ≤ 0.001). The Klebsiella abundance was strongly negatively correlated with body weight, and strongly positively correlated with GLU, INS, the HOMA-IR index, TG, ANG-2, MAP, α-HBDH, CK-MB, LDH, IL-6 (*p* ≤ 0.001). This result indicated that there is a close and complicated connection between gut microbiota and host health.

## 4. Discussion

Because of the prevalence of a high-calorie dietary style and other unhealthy living habits, T2D is a growing epidemic worldwide. T2D, together with its complications such as cardiovascular and cerebrovascular diseases, contributes to high rates of morbidity and mortality [17]. Currently, there is an urgent and recognized need for exploring plant-derived natural products to protect against the risks of T2D and its complications. RA is a major triterpene constituent with extensive bioactivities in the barks of *Ilex rotunda* Thunb. [10,11], which are widely used to make herbal teas for health care in southern China. In this study, the protective effects of RA on metabolic disturbance and imbalance of gut microbiota were investigated in T2D rats. Our findings first provide direct evidence showing that RA is a potential nutraceutical agent to protect against T2D and improve gut microbiota.

In the present study, we employed a combination of HFD feeding and a single low-dose STZ injection to mimic human T2D characteristics, including hyperglycemia, dyslipidemia and hyperinsulinemia [18]. HFD feeding resulted in obesity first at week 6 and STZ injection then caused continuous weight loss after week 8 as expected. The body weights of T2D patients commonly continue to reduce with the development of T2D, particularly when the T2D patients had inadequate blood glucose control [19]. However, T2D patients with a low body mass index (BMI) are more likely to suffer from a higher total fat mass and abdominal obesity compared with a BMI-matched normal control group, and weight loss without an intrusive body weight control strategy implies increased glycemic variability and risks of diabetic complications [20,21]. RA treatment alleviated weight loss, and abnormal food and water intake, suggesting that it could help improve the physical states of T2D rats. Effective control of the blood glucose level is a key step in preventing or reversing diabetic complications and improving the quality of life in T2D patients [22]. RA treatment resulted in a significant decrease in GLU, INS and HbAc1 level, and insulin resistance according to the HOMA-IR index, OGTT and ITT, indicating its significant hypoglycemic activity. T2D is also characterized by dyslipidemia, which is a risk factor of cardiovascular and cerebrovascular diseases. RA treatment could improve lipid metabolism in terms of the serum levels of TG, TC, LDL-C, HDL-C and FFAs. Oxidative stress and low-grade inflammation may cause insulin resistance and impair pancreatic β-cells in the T2D progression and are important risk factors of cardiovascular and cerebrovascular diseases [23]. Notably, altered plasma oxidative stress factors and inflammatory marker concentrations were observed in T2D patients with low BMI compared to obese T2D patients, which can induce the insulin resistance and pancreatic β-cell apoptosis [24]. The CD4+CXCR5+ T cells from T2D patients with low BMI were highly enriched in IFN-γ-producing cells and depleted in IL-4- and IL-17-producing cells [25]. Although T2D with low body weight is most often linked to pancreatic β-cell dysfunction, oxidative stress and intestinal inflammation may also play important roles in it [23,24]. These features of T2D patients with low BMI described above were also evidently observed in the T2D model groups of our experiment. The turbulences of serum oxidative stress factors (SOD, MDA) and cytokine (TNF-α, INF-γ, IL-1β, IL-4, and IL-6) were regulated by RA treatment, suggesting RA could protect against oxidative stress and low-grade inflammation in T2D. It is likely that the regular effects of RA on oxidative stress factors and cytokine are associated with intestinal inflammation and gut microbiota.

Cardiovascular diseases are common diabetic complications, with high rates of mortality and morbidity. Serum myocardial enzymes (α-HBDH, CK, CK-MB and LDH) and CRP levels were always evaluated as key indicators of cardiovascular injury in clinal practice. the serum myocardial enzymes and CRP levels proved that RA treatment may play a positive role in protecting the risk of serious cardiovascular diseases. In addition, RA treatment could exert potent hypertension-lowering effects due to reduced SYS, DIA and MAP levels compared with the T2D model group. ET-1 is increasingly secreted by damaged vascular endothelial cell, which actively involves in the pathogenic processes of hypertension, vascular remodeling, endothelial dysfunction and inflammation [26]. ANG-2 is another crucial participant in modulating vascular functions and inflammation via the renin–angiotensin system [27]. RA treatment significantly down-regulated the elevated levels of ET-1 and ANG-2 in T2D rats, suggesting that its protective effects against vascular injury and vasoconstriction. As for hepatic and renal injury, the abnormally high serum levels of ALT, AST, ALP, UA and BUN in the T2D rats were significantly decreased by RA treatment, which is obviously due to the decreased hepatocyte and nephrocyte necrosis induced by lipotoxicity and glucotoxicity.

Triterpene is an important ingredient of medicinal and food plant, which commonly has extensive bioactivities but low bioavailability [28]. Moreover, a previous report showed that RA could be metabolized into bioactive metabolites by microbial metabolism [12,13]. Therefore, we hypothesized that the protective effects of RA against T2D were partly explained by its ability to improve gut microbiota and 16 s rRNA gene amplification and sequencing were conducted in T2D rats. Consistent with other reports, remarkable turbulence of gut microbiota was demonstrated to be associated with T2D. Microbiota richness and diversity are essential to maintain ecosystem stability and efficiency and other studies suggested that low microbiota richness diversity may be associated with various diseases including diabetes and atherosclerosis [7]. According to α-diversity analysis, a lower Chao1 value and Shannon value were observed in T2D model group in comparison with the control group, indicating low microbiota richness and diversity with T2D. Notably, the decrease in microbiota richness and diversity was ameliorated by RA treatment, which might benefit to improve metabolic disturbance in T2D. In addition, PCoA analysis based on weighted unifrac metrics displayed the discriminative clusters representing microbial communities of three tested groups, confirming that RA treatment could significantly alter abnormal microbial community in T2D. At the phylum level, the T2D model group had an elevated abundance of *Firmicutes* and a reduced abundance of *Bacteroidetes* compared with the control group. A high ratio of *Firmicutes* to *Bacteroidetes* was also found in patients and rats with obesity, diabetes and coronary artery disease in previous studies [29]. The imbalance at the phylum level was a common risk factor of various health problems and RA treatment had a tendency of restoring it. At the genus level, the T2D model group had lower beneficial or commensal bacteria, such as *Paraprevotella*, *Oscillospira*, *Turicibacter*, *Prevotella* and *Ruminococcus*, and more opportunistic pathogens such as *Blautia*, *Allobaculum*, *Klebsiella*, *Proteus*, *Christensenella*, *Dorea*, *Butyricicoccus*, *Citrobacter*, *Coprococcus* and *Clostridium* compared with the control group. For instance, *Oscillospira* is able to utilize host glycans as growth substrates and decreases in abundance in obesity and inflammatory diseases [30]. It is implied that hyperglycemia and low-grade inflammation are associated with decreased *Oscillospira* abundance in the T2D model group. A previous study reported that *Turicibacter* was positively correlated with butyric acid, which was considered as a beneficial substance in the intestinal tract [31]. A high ratio of Blautia in diabetic children or obesity mice have also been reported in recent investigations and could be restore to a certain extent by some functional food such as the extract of *Chlorella pyrenoidosa* [32,33]. These findings indicated that the roles of numerous gut bacteria were diverse and complex in the disease progression.

Gut microbiota might be a possible target of RA for attenuation of T2D. *Klebsiella* and *Proteus* both belong to *Enterobacteriaceae* and induce various infectious diseases such as hepatic abscess, necrotizing fasciitis and urosepsis associated with diabetes [34,35]. As Gram-negative bacteria, most members of *Klebsiella* and *Proteus* are lipopolysaccharide (LPS) producers and were found to be associated with diabetes and cerebral ischemic stroke [36,37]. LPS can activate pro-inflammatory cytokine production resulting in obesity and impaired glucose tolerance [38]. Consistently, RA treatment decreased *Klebsiella* and *Proteus* abundance in T2D rats. *Klebsiella* and *Proteus* abundance was significantly positively correlated with GLU, INS, the HOMA-IR index, TG, ANG-2, MAP, α-HBDH, CK-MB, LDH, TNF-α, IL-1β and IL-6, suggesting that the inhibitive effects of RA may help improve glycolipid metabolism, cardiovascular function and inflammation under the diabetic condition. Previous studies reported that *Prevotella* was less abundant in T2D rats and that the increased *Prevotella* abundance was associated with the beneficial functions of some health food such as barley kernels. It is probably because *Prevotella* could produce short-chain fatty acids (SCFAs) in the intestinal tract and promotes increased hepatic glycogen storage in mice [39,40]. Interestingly, the *Prevotella* abundance was increased by RA treatment, which was strongly negatively correlated with INS, TC, α-HBDH, CK, LDH and CRP, and strongly positively correlated with SOD. In addition, the *Ruminococcus* abundance was significantly increased by RA treatment. *Ruminococcus* is a bacterial genus belonging to *Ruminococcaceae* with unique metabolism features including the production of butyrate from both sugars and amino acids. Butyrate is a main component of SCFAs that can provide an energy substrate to colonocytes, mitigate inflammation, and regulate satiety, etc. [41]. Deficiency in SCFA production has been associated with diseases, including T2D, and butyrate supplementation prevented insulin resistance and obesity in mice [32,42]. These results suggested RA treatment might increase *Ruminococcus* abundance to alleviate metabolic disturbance in T2D rats via promoting SCFA production in the intestinal tract. Moreover, we also found that RA treatment could increase *Leuconostoc* and *Streptococcus* abundances in gut microbiota. *Leuconostoc* and *Streptococcus* are a series of bacteria called lactic acid bacteria belonging to *Lactobacillaceae*. As the major probiotic, lactic acid bacteria are considered to be key bacteria that benefit the host health [29]. Lactic acid bacteria produce lactic acid, CO_2_, acetic acid, and/or ethanol from carbohydrate, which contribute to a more acidic environment in the intestinal tract [43]. Various lactic acid bacteria have been related with an improvement in glucose metabolism and a reduction in chronic inflammation and have been found in reduced quantities in patients with T2D and obesity [44,45]. However, the current studies are still limited and the direct molecular mechanisms explaining relationships between gut microbiota and host health need further study.

## 5. Conclusions

In summary, we demonstrated that RA has multipronged effects on metabolic disturbance in T2D using a T2D rat model induced by HFD feeding and a single low-dose STZ injection, including improving glucolipid metabolism, lowering blood pressure, protecting against cardiovascular and hepatorenal injuries, and suppressing oxidative stress and inflammation. Furthermore, we first provided direct evidence showing that RA could alleviate gut microbiota dysbiosis in T2D by 16s rRNA gene V4–V5 region amplification and sequencing, and that the compositions of gut microbiota were strongly correlative with multiple biochemical parameters. Our results support RA as a nutraceutical agent or plant foods rich in this compound might be helpful for the alleviation of T2D and its complications through improving gut microbiota.

## Figures and Tables

**Table 1:** Serum myocardial enzymes (α-HBDH, CK, CK-MB and LDH), CRP and ET-1 levels of the control group, T2D model group and RA-treated group (40 mg/kg/day) at the end of the experiment.

	Control	T2D Model	RA-Treated
α-HBDH (U/L)	104.8 ± 12.5	525.1 ± 37.1 **	151.9 ± 19.6 ##
CK (U/L)	0.230 ± 0.028	0.712 ± 0.095 **	0.418 ± 0.090 ##
CK-MB (U/L)	164.9 ± 12.5	474.5 ± 45.2 **	191.7 ± 27.9 ##
LDH (U/L)	1405.7 ± 175.0	2352.1 ± 148.1 **	1854.0 ± 163.8 ##
CRP (pg/mL)	305.3 ± 32.2	647.3 ± 29.6 **	422.7 ± 46.2 ##
ET-1 (pg/mL)	33.1 ± 5.9	61.5 ± 3.3 **	48.9 ± 12.7 #
ALT (IU/L)	22.7 ± 9.2	111.7 ± 18.3 **	44.6 ± 13.4 ##
AST (IU/L)	92.5 ± 13.4	174.7 ± 23.8 **	99.32 ± 12.6 ##
ALP (KU/100 mL)	11.4 ± 2.3	53.4 ± 4.2 **	15.1 ± 3.0 ##
UA (μmol/L)	66.2 ± 16.0	208.9 ± 35.2 **	131.0 ± 38.4 ##
BUN (mmol/L)	4.9 ± 0.7	10.8 ± 1.3 **	5.3 ± 1.1 ##
CRE (μmol/L)	42.2 ± 7.0	62.6 ± 12.6 **	52.9 ± 4.6

Data are present as the mean ± SD, *n* = 8. ** *p* < 0.01 vs. control group; # *p* < 0.05 vs. T2D model group; ## *p* < 0.01 vs. T2D model group by Tukey’s multiple comparison test.

**Table 2:** Oxidative stress factors (SOD, MDA) and cytokines (TNF-α, INF-γ, IL-1β, IL-4, and IL-6) of the control group, T2D model group and RA-treated group (40 mg/kg/day) at the end of the experiment.

	Control	T2D Model	RA-Treated
SOD (U/L)	269.2 ± 20.5	216.3 ± 19.6 **	246.7 ± 10.6 ##
MDA (nmol/mL)	4.29 ± 0.63	6.20 ± 0.59 **	4.73 ± 0.93 ##
TNF-α (pg/mL)	234.9 ± 23.4	566.5 ± 72.6 **	259.1 ± 43.2 ##
INF-γ (pg/mL)	18.9 ± 3.9	23.0 ± 4.1	16.6 ± 1.7 ##
IL-1β (pg/mL)	9.6 ± 3.0	19.0 ± 2.7 **	11.0 ± 1.7 ##
IL-4 (pg/mL)	54.0 ± 5.9	37.1 ± 4.0 **	55.2 ± 10.6 ##
IL-6 (pg/mL)	6.5 ± 1.2	14.4 ± 2.5 **	8.7 ± 1.7 ##

Data are present as the mean ± SD, *n* = 8. ** *p* < 0.01 vs. control group; ## *p* < 0.01 vs. T2D model group by Tukey’s multiple comparison test.

## Supplementary Materials

The following are available online at https://www.mdpi.com/2072-6643/12/1/67/s1, Figure S1: UHPLC-UV chromatograms of the RA sample (A) and the RA standard (B), raw sequence data in this study are deposited in Sequence Read Archive (SRA) (https://www.ncbi.nlm.nih.gov/sra/PRJNA587396) with the accession number PRJNA587396.

## References

[1] Weber K.S., Nowotny B., Strassburger K., Pacini G., Mussig K., Szendroedi J., Herder C., Roden M. (2015). The Role of Markers of Low-Grade Inflammation for the Early Time Course of Glycemic Control, Glucose Disappearance Rate, and beta-Cell Function in Recently Diagnosed Type 1 and Type 2 Diabetes. Diabetes Care.

[2] Cho N.H., Shaw J.E., Karuranga S., Huang Y., da Rocha Fernandes J.D., Ohlrogge A.W., Malanda B. (2018). IDF Diabetes Atlas: Global estimates of diabetes prevalence for 2017 and projections for 2045. Diabetes Res. Clin. Pract..

[3] Evans J.M., Newton R.W., Ruta D.A., MacDonald T.M., Morris A.D. (2000). Socio-economic status, obesity and prevalence of Type 1 and Type 2 diabetes mellitus. Diabet. Med. J. Br. Diabet. Assoc..

[4] Caspard H., Jabbour S., Hammar N., Fenici P., Sheehan J.J., Kosiborod M. (2018). Recent trends in the prevalence of type 2 diabetes and the association with abdominal obesity lead to growing health disparities in the USA: An analysis of the NHANES surveys from 1999 to 2014. Diabetes Obes. Metab..

[5] Chiriaco M., Pateras K., Virdis A., Charakida M., Kyriakopoulou D., Nannipieri M., Emdin M., Tsioufis K., Taddei S., Masi S. (2019). Association between blood pressure variability, cardiovascular disease and mortality in type 2 diabetes: A systematic review and meta-analysis. Diabetes Obes. Metab..

[6] Qin J., Li Y., Cai Z., Li S., Zhu J., Zhang F., Liang S., Zhang W., Guan Y., Shen D. (2012). A metagenome-wide association study of gut microbiota in type 2 diabetes. Nature.

[7] Sanchez-Alcoholado L., Castellano-Castillo D., Jordan-Martinez L., Moreno-Indias I., Cardila-Cruz P., Elena D., Munoz-Garcia A.J., Queipo-Ortuno M.I., Jimenez-Navarro M. (2017). Role of Gut Microbiota on Cardio-Metabolic Parameters and Immunity in Coronary Artery Disease Patients with and without Type-2 Diabetes Mellitus. Front. Microbiol..

[8] Kim Y., Keogh J., Clifton P. (2015). A review of potential metabolic etiologies of the observed association between red meat consumption and development of type 2 diabetes mellitus. Metab. Clin. Exp..

[9] Zhao L., Zhang F., Ding X., Wu G., Lam Y.Y., Wang X., Fu H., Xue X., Lu C., Ma J. (2018). Gut bacteria selectively promoted by dietary fibers alleviate type 2 diabetes. Science.

[10] He Y.F., Nan M.L., Sun J.M., Meng Z.J., Yue F.G., Zhao Q.C., Yang X.H., Wang H. (2012). Synthesis, characterization and cytotoxicity of new rotundic acid derivatives. Molecules.

[11] Hsu Y.M., Hung Y.C., Hu L., Lee Y.J., Yin M.C. (2015). Anti-Diabetic Effects of Madecassic Acid and Rotundic Acid. Nutrients.

[12] Li H., Yang B., Cao D., Zhou L., Wang Q., Rong L., Zhou X., Jin J., Zhao Z. (2018). Identification of rotundic acid metabolites after oral administration to rats and comparison with the biotransformation by Syncephalastrum racemosum AS 3.264. J. Pharm. Biomed. Anal..

[13] Yang B., Li H., Ruan Q., Xuan S., Chen X., Cui H., Liu Z., Jin J., Zhao Z. (2019). Effects of Gut Microbiota and Ingredient-Ingredient Interaction on the Pharmacokinetic Properties of Rotundic Acid and Pedunculoside. Planta Med..

[14] Edgar R.C. (2013). UPARSE: Highly accurate OTU sequences from microbial amplicon reads. Nat. Methods.

[15] Caporaso J.G., Kuczynski J., Stombaugh J., Bittinger K., Bushman F.D., Costello E.K., Fierer N., Pena A.G., Goodrich J.K., Gordon J.I. (2010). QIIME allows analysis of high-throughput community sequencing data. Nat. Methods.

[16] Wang Q., Garrity G.M., Tiedje J.M., Cole J.R. (2007). Naive Bayesian classifier for rapid assignment of rRNA sequences into the new bacterial taxonomy. Appl. Environ. Microbiol..

[17] Zheng Y., Ley S.H., Hu F.B. (2018). Global aetiology and epidemiology of type 2 diabetes mellitus and its complications. Nat. Rev. Endocrinol..

[18] Guo X.X., Wang Y., Wang K., Ji B.P., Zhou F. (2018). Stability of a type 2 diabetes rat model induced by high-fat diet feeding with low-dose streptozotocin injection. J. Zhejiang Univ. Sci. B.

[19] Expert Committee on the Diagnosis and Classification of Diabetes Mellitus (2003). Report of the expert committee on the diagnosis and classification of diabetes mellitus. Diabetes Care.

[20] Wang J., Yan R., Wen J., Kong X., Li H., Zhou P., Zhu H., Su X., Ma J. (2017). Association of lower body mass index with increased glycemic variability in patients with newly diagnosed type 2 diabetes: A cross-sectional study in China. Oncotarget.

[21] Yang S., Wang S., Yang B., Zheng J., Cai Y., Yang Z. (2016). Weight loss before a diagnosis of type 2 diabetes mellitus is a risk factor for diabetes complications. Medicine.

[22] Mo F.F., Lv B.H., An T., Miao J.N., Liu J.X., Zhang J., Zhang Z.Y., Ma M.H., Yang X.Y., Zhao D. (2019). Protective mechanism of punicalagin against endoplasmic reticulum stress in the liver of mice with type 2 diabetes mellitus. J. Funct. Foods.

[23] Hansen D., Dendale P., Beelen M., Jonkers R.A., Mullens A., Corluy L., Meeusen R., van Loon L.J. (2010). Plasma adipokine and inflammatory marker concentrations are altered in obese, as opposed to non-obese, type 2 diabetes patients. Eur. J. Appl. Physiol..

[24] Chan W.B., Tong P.C., Chow C.C., So W.Y., Ng M.C., Ma R.C., Osaki R., Cockram C.S., Chan J.C. (2004). The associations of body mass index, C-peptide and metabolic status in Chinese Type 2 diabetic patients. Diabet. Med. J. Br. Diabet. Assoc..

[25] Zhou J., Wang Y., He Y., Gao Y., Wan R., Cai M., Li W., Chen R., Walker E., Zhai X. (2018). Non-obese type 2 diabetes patients present intestinal B cell dysregulations associated with hyperactive intestinal Tfh cells. Mol. Immunol..

[26] Shihoya W., Nishizawa T., Okuta A., Tani K., Dohmae N., Fujiyoshi Y., Nureki O., Doi T. (2016). Activation mechanism of endothelin ETB receptor by endothelin-1. Nature.

[27] Siddiqui K., Joy S.S., Nawaz S.S., Al Otaibi M.T., Al-Rubeaan K. (2018). Angiopoietin-2 level as a tool for cardiovascular risk stratification in hypertensive type 2 diabetic subjects. Postgrad. Med..

[28] Santangelo R., Silvestrini A., Mancuso C. (2019). Ginsenosides, catechins, quercetin and gut microbiota: Current evidence of challenging interactions. Food Chem. Toxicol..

[29] Zhang Q., Yu H., Xiao X., Hu L., Xin F., Yu X. (2018). Inulin-type fructan improves diabetic phenotype and gut microbiota profiles in rats. PeerJ.

[30] Konikoff T., Gophna U. (2016). Oscillospira: A Central, Enigmatic Component of the Human Gut Microbiota. Trends Microbiol..

[31] Zhong Y., Nyman M., Fak F. (2015). Modulation of gut microbiota in rats fed high-fat diets by processing whole-grain barley to barley malt. Mol. Nutr. Food Res..

[32] Wan X.Z., Li T.T., Zhong R.T., Chen H.B., Xia X., Gao L.Y., Gao X.X., Liu B., Zhang H.Y., Zhao C. (2019). Anti-diabetic activity of PUFAs-rich extracts of Chlorella pyrenoidosa and Spirulina platensis in rats. Food Chem. Toxicol..

[33] Zheng J., Zhang J., Guo Y., Cui H., Lin A., Hu B., Gao Q., Chen Y., Liu H. (2020). Improvement on metabolic syndrome in high fat diet-induced obese mice through modulation of gut microbiota by sangguayin decoction. J. Ethnopharmacol..

[34] Lee C.C., Lee C.H., Hong M.Y., Hsieh C.C., Tang H.J., Ko W.C. (2018). Propensity-matched analysis of the impact of extended-spectrum beta-lactamase production on adults with community-onset Escherichia coli, Klebsiella species, and Proteus mirabilis bacteremia. J. Microbiol. Immunol. Infect..

[35] Ho P.L., Tang W.M., Yuen K.Y. (2000). Klebsiella pneumoniae necrotizing fasciitis associated with diabetes and liver cirrhosis. Clin. Infect. Dis..

[36] Chen R., Wu P., Cai Z., Fang Y., Zhou H., Lasanajak Y., Tang L., Ye L., Hou C., Zhao J. (2019). Puerariae Lobatae Radix with chuanxiong Rhizoma for treatment of cerebral ischemic stroke by remodeling gut microbiota to regulate the brain-gut barriers. J. Nutr. Biochem..

[37] Wirth R., Bodi N., Maroti G., Bagyanszki M., Talapka P., Fekete E., Bagi Z., Kovacs K.L. (2014). Regionally distinct alterations in the composition of the gut microbiota in rats with streptozotocin-induced diabetes. PLoS ONE.

[38] Cani P.D., Bibiloni R., Knauf C., Waget A., Neyrinck A.M., Delzenne N.M., Burcelin R. (2008). Changes in gut microbiota control metabolic endotoxemia-induced inflammation in high-fat diet-induced obesity and diabetes in mice. Diabetes.

[39] Kovatcheva-Datchary P., Nilsson A., Akrami R., Lee Y.S., De Vadder F., Arora T., Hallen A., Martens E., Bjorck I., Backhed F. (2015). Dietary Fiber-Induced Improvement in Glucose Metabolism Is Associated with Increased Abundance of Prevotella. Cell Metab..

[40] Ding Q., Zhang B., Zheng W., Chen X., Zhang J., Yan R., Zhang T., Yu L., Dong Y., Ma B. (2019). Liupao tea extract alleviates diabetes mellitus and modulates gut microbiota in rats induced by streptozotocin and high-fat, high-sugar diet. Biomed. Pharmacother..

[41] Nicholson J.K., Holmes E., Kinross J., Burcelin R., Gibson G., Jia W., Pettersson S. (2012). Host-gut microbiota metabolic interactions. Science.

[42] Zhao F., Liu Q., Cao J., Xu Y., Pei Z., Fan H., Yuan Y., Shen X., Li C. (2019). A sea cucumber (Holothuria leucospilota) polysaccharide improves the gut microbiome to alleviate the symptoms of type 2 diabetes mellitus in Goto-Kakizaki rats. Food Chem. Toxicol..

[43] Hemme D., Foucaud-Scheunemann C. (2004). Leuconostoc, characteristics, use in dairy technology and prospects in functional foods. Int. Dairy J..

[44] Díaz-Rizzolo D.A., Kostov B., López-Siles M., Serra A., Colungo C., González-de-Paz L., Martinez-Medina M., Sisó-Almirall A., Gomis R. (2019). Healthy dietary pattern and their corresponding gut microbiota profile are linked to a lower risk of type 2 diabetes, independent of the presence of obesity. Clin. Nutr..

[45] Lee S., Kim M. (2019). Leuconostoc mesenteroides MKSR isolated from kimchi possesses α-glucosidase inhibitory activity, antioxidant activity, and cholesterol-lowering effects. LWT.

