# Supplementary material for: Rotundic Acid Protects against Metabolic Disturbance and Improves Gut Microbiota in Type 2 Diabetes Rats
Source: Nutrients. 2019 Dec 26;12(1):67. doi: 10.3390/nu12010067 (PMC7019423; doi:10.3390/nu12010067)
Supplement: Supplementary file 1 [file nutrients-12-00067-s001.pdf]

Rotundic acid protects against metabolic disturbance and improves gut microbiota in type 2 diabetes rats

Zenghao Yan [1], Hao Wu [1], Hongliang Yao [1,2,3], Wenjun Pan [1], Minmin Su [1], Taobin Chen [1,2], Weiwei Su [1] and Yonggang Wang [1,*]

[1] State Key Laboratory of Biocontrol and Guangdong Provincial Key Laboratory of Plant Resources, School of Life Sciences, Sun Yat-sen University, Guangzhou, Guangdong, 510275, China; yanzengh@mail2.sysu.edu.cn (Z.Y.); wuhao8@mail.sysu.edu.cn (H.W.); yaohl@giabr.gd.cn (H.Y.); panwj23@mail2.sysu.edu.cn (W.P.); tagorem@163.com (M.S.); sysulssctb@126.com (T.C.); lssswww@126.com (W.S.)

[2] Shenzhen Research Institute of Sun Yat-sen University, Shenzhen, Guangdong, 518057, China

[3] Guangdong Key Laboratory of Animal Conservation and Resource Utilization, Guangdong Public Laboratory of Wild Animal Conservation and Utilization, Drug Synthesis and Evaluation Center, Guangdong Institute of Applied Biological Resources, Guangzhou, Guangdong, 510260, China

* Correspondence: wangyg@mail.sysu.edu.cn; Tel: +86-20-8411-1288 (Y.W.)

Supplementary materials

In order to confirm the purity and structure of rotundic acid (RA), a qualitative and quantitative analysis was performed using UHPLC-UV. The UHPLC-UV analysis was conducted on a Dionex U3000 UHPLC system (LPG-3400SD pumps, WPS-3000 sampler, TCC-3000 column oven and DAD-3000 detector, Thermo Fisher Scientific, Waltham, USA). LC separation was performed on a 5 μm 4.6 × 250 mm ZORBAX Eclipse Pluc C_{18} column (Agilent, Santa Clara, USA) at 30 °C with a flow rate of 1 mL/min. The elution gradient program with solvent A (deionized water with 0.1% formic acid) and solvent B (acetonitrile with 0.1% formic acid) was set as follows: 0-15 min, isocratic 33% B; 15-20 min linear gradient from 33% to 50% B; 20-30 min isocratic 50% B, and afterward back to 33% B in 10 min. The UV detection wavelength and injection volume was 210 nm and 10 μL, respectively. As shown in Figure S1, RA sample prepared in this study was verified by RA standard and the purity was ＞ 98%.

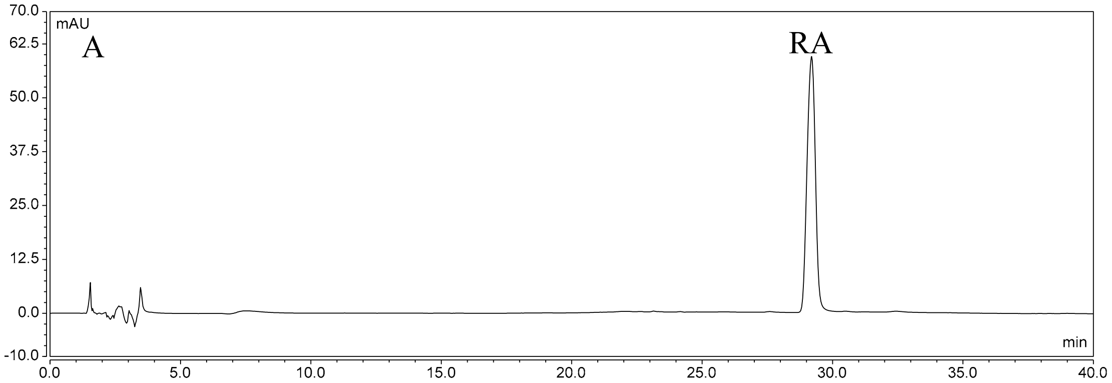

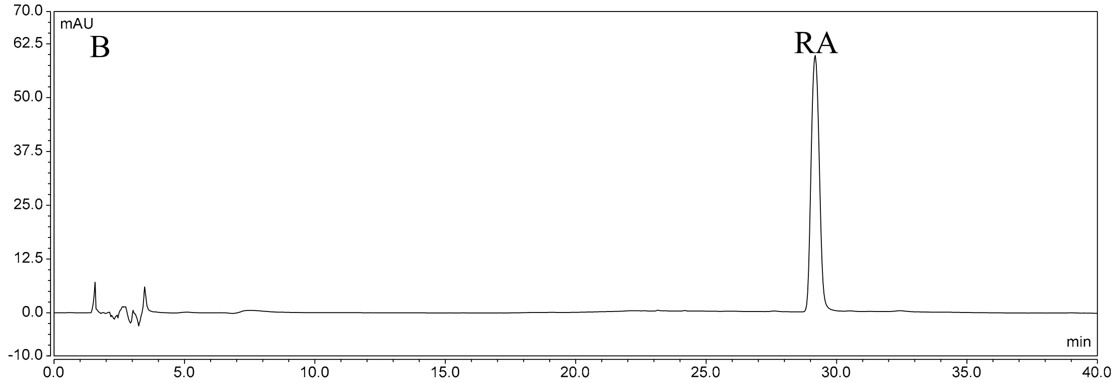

Figure S1. UHPLC-UV chromatograms of the RA sample (A) and the RA standard (B).
